# Patient and Staff Perspectives on a Non-Restrictive Leave Protocol at Springbank Ward, Specialist Personality Disorder Unit

**DOI:** 10.1192/bjo.2022.101

**Published:** 2022-06-20

**Authors:** Adelaide Yue, Owen Crawford, Jorge Zimbron

**Affiliations:** 1 University of Cambridge, Cambridge, United Kingdom; 2 Sandwell and West Birmingham Hospitals NHS Trust, Birmingham, United Kingdom; 3 Cambridgeshire and Peterborough NHS Foundation Trust, Cambridge, United Kingdom

## Abstract

**Aims:**

Springbank Ward is a specialist unit for patients with a diagnosis of emotionally unstable personality disorder (EUPD). Psychiatric wards often use restrictive practices to try and minimise suicide risk. Using risk assessment checklists to decide whether to grant leave is one example. Research shows that it is not possible to predict suicide at an individual level, regardless of the assessment method used, so we questioned the utility of such an approach. A previous evaluation of our leave protocol showed that patients and staff would favour a less restrictive and more personalised approach. We introduced a new protocol that eliminated use of checklists, replacing them with an optional 1:1 conversation with staff before leaving the ward. Our aim was to gauge patient and staff satisfaction with the new protocol and investigate their views on the change.

**Methods:**

Data were obtained through structured interviews with staff who assessed risk (nurses and psychiatrists) and patients. 9 patients and 8 members of staff were interviewed between 9–19 March 2021. Interviewees were presented with diagrams of both the new protocol and old risk assessment checklist and asked a series of questions, including: rating their satisfaction; any potential improvements; and whether they would prefer the previous or current protocol. Thematic analysis of interview answers was used to explore patient and staff perspectives. Two authors independently analysed the interview transcripts, before discussing any discrepancies to reach a unified set of themes, subthemes and codes.

**Results:**

Both patients and staff gave the new protocol an average satisfaction rating of 4.1/5. Thematic analysis generated five themes: “taking ownership”, “autonomy Vs restriction”, “staff-patient interaction”, “staff expertise” and “protocol efficiency”. Most interviewees agreed that the new protocol supported patients in taking responsibility for their safety, helping to prepare for life in the community. The protocol was considered minimally restrictive and more efficient than the previous system. The importance of communication and trust between patients and staff, as well as the use of staff intuition in holistically assessing risk, was emphasised. Potential disadvantages included the perceived riskiness of reducing restrictions and difficulty seeking support early in the admission.

**Conclusion:**

In general, the new protocol is rated highly by patients and staff and is considered to be minimally restrictive and more holistic, in line with the aims established by our previous evaluation. Our findings have implications regarding risk management for inpatients with EUPD.

